# Depression and Associated Factors Among Patients with Spinocerebellar Ataxia

**DOI:** 10.3390/medicina61010160

**Published:** 2025-01-19

**Authors:** Sirivipa Jekpoo, Nahathai Wongpakaran, Tinakon Wongpakaran, Chayasak Wantaneeyawong, Punjaree Wiriyacosol, Pised Methapatara

**Affiliations:** 1 Department of Psychiatry, Faculty of Medicine, Chiang Mai University, Chiang Mai 50200, Thailand; sirivipa.jek@cmu.ac.th (S.J.); tinakon.w@cmu.ac.th (T.W.);; 2 Department of Internal Medicine, Faculty of Medicine, Chiang Mai University, Chiang Mai 50200, Thailand; chayasak.w@cmu.ac.th; 3 Suanprug Hospital, Chang Wat Ratchaburi 70180, Thailand; pisedme@gmail.com

**Keywords:** spinocerebellar ataxia, depression, anxiety, mental health

## Abstract

*Background and Objectives:* Spinocerebellar ataxia (SCA) is a progressive neurodegenerative disease often accompanied by depression. This cross-sectional study investigated the prevalence of depression and the associated mental health factors in SCA patients. *Material and Methods:* Eleven Thai SCA patients completed questionnaires assessing depression, anxiety, inner strengths, perceived social support, personality traits and perceived stress. *Results:* Participants’ average age was 50.27 years old. The prevalence of depression was 27.27%. Depression scores were positively correlated with OI-anxiety score (*r* = 0.887, 95%CI 0.586 to 0.968), perceived stress (*r* = 0781, 95%CI 0.305 to 0.936) and personality traits including aggression (*r* = 0.73, 95% CI 0.197 to 0.920), activity (*r* = 0.651, 95%CI 0.052 to 0.893) and neuroticism (*r* = 0.80, 95% CI 0.351 to 0.942). Conversely, depression negatively correlated with inner strengths (*r* = −0.70, 95%CI −0.910 to −0.139) and perceived social support, particularly from family (*r* = −0.88, 95%CI −0.966 to −0.564). *Conclusions:* These findings highlight the need for comprehensive mental health assessment and intervention in SCA patients. Strengthening inner strengths, promoting social support, and managing negative mental health factors may improve quality of life for patients with SCA.

## 1. Introduction

Spinocerebellar ataxia (SCA) is a degenerative disorder affecting the cerebellum and is inherited as an autosomal dominant disorder. The worldwide prevalence of SCA is estimated to be between 1 and 5 cases per 100,000 persons [1]. A recent systematic review highlighted the scarcity of evidence-based interventions specifically for SCA [2], resulting in inevitable dependency for affected patients. Ataxic symptoms significantly increase the risk of falls for patients with SCA and adversely affect their ability to function, including factors such as self-care, mobility and movement [3]. Most patients require the use of a wheelchair within 10 to 15 years following the onset of symptoms. The average 10-year survival rates are approximately 57% for SCA type 1, 74% for SCA type 2, 73% for SCA type 3 and 87% for SCA type 6 [4]. In addition to physical symptoms, non-motor symptoms such as fatigue, poor sleep quality, cognitive impairment and depression may also manifest [5,6,7]. While the prevalence of depression is 38% in patients with Parkinson’s disease [8], which also has motor symptoms, the prevalence of depression in SCAs is 13% to 69% [5]. Depression among patients with SCA is partially attributed to neurodegeneration, as the cerebellum is extensively connected to various regions of the frontal lobe and brainstem. Consequently, degeneration of the cerebellum can lead to cognitive deficits and mood disturbances, known as cerebellar cognitive affective syndrome [9]. Depression has a profound impact on life and social functioning; it may also diminish treatment adherence, increase disease burden and lead to a poorer prognosis [9,10].

Related studies have examined mental health factors associated with depression, and found that perceived social support from family and friends correlates with reduced depressive symptoms among depressed patients [11]. This finding aligns with research on patients with amyotrophic lateral sclerosis (ALS), revealing that depression among patients with ALS was negatively associated with social support and environmental resources [12]. Furthermore, a study of inner strengths revealed that some psychological strengths, such as equanimity, were associated with reduced depression [13].

Conversely, research on depression among patients with SCA has identified a correlation between depression and anxiety [14]. Studies examining stress levels among patients with incurable chronic diseases have shown that elevated stress is associated with increased depression [15]. Additionally, research into personality traits linked to depression indicates that neuroticism personality traits are correlated with greater severity of depressive symptoms [16], suggesting that a high level of anxiety increases the risk of depression [17].

Moreover, the progression of the disease is associated with increased levels of depression. A study using a self-reported online questionnaire on mental health among patients with SCA found that a greater severity of ataxia symptoms is associated with higher levels of depression and anxiety [18]. Additionally, more severe ataxia symptoms are associated with poorer sleep quality and greater cognitive impairment [14,19].

Related research has explored various mental health factors associated with depression in the general population or other chronic diseases, such as perceived social support [11], inner strengths [13], anxiety [14], perceived stress [11] and personality traits [16]. Moreover, these factors, particularly social support, have been linked to depression among patients with ALS, another neurodegenerative disease [12]. There remains a significant gap in the knowledge regarding the specific impact of these factors on depression among patients with SCA. 

From these literature reviews, it is evident that mental health factors contribute to the development of depression, poor sleep quality, and cognitive impairment. However, there remains a significant gap in the knowledge regarding the mental health factors specifically associated with depression among patients with spinocerebellar ataxia. Therefore, this study has two primary objectives, as stated below.

1. To investigate the prevalence of depression in patients with SCA.
2. To examine the relationship between positive and negative mental health factors and depression in patients with SCA.

By achieving these objectives, this study aims to contribute to a better understanding of the mental health challenges faced by patients with SCA and contribute to developing effective interventions to improve their well-being.

## 2. Materials and Methods

### 2.1. Study Design

This study constitutes a cross-sectional study of 11 Thai patients with SCA at Maharaj Nakhon Chiang Mai Hospital who were recruited to participate in the study. Ethical review and approval were obtained from the Faculty of Medicine, Chiang Mai University, Thailand (study code, PSY-2566-0528).

### 2.2. Participants and Setting

The eligibility criteria are described below.

1. Diagnosis of SCA using ICD-10 codes G11.1 (early-onset cerebellar ataxia) or G11.2 (late-onset cerebellar ataxia).
2. Aged 20 years or older. This criterion was implemented to focus on the adult manifestation of SCA and to minimize the potential influence of developmental factors on mental health.
3. Thai ethnicity. This criterion was included to control cultural factors influencing mental health experiences and to help ensure that the assessment tools were culturally appropriate.
4. Fluency in the Thai. This ensured that participants could fully understand the assessment tools and provide accurate responses.

Patients with unstable vital signs, serious contagious diseases, infection in the spreading stage and those receiving a diagnosis of other hereditary or secondary ataxias were excluded.

The participant assessment in this study was conducted in two formats: face-to-face or online for patients unable to come to the hospital. Participants signed informed consent forms before being included in the study. All participants provided information on the following: demographics, previous psychiatric history, family history of psychiatric disease and age at onset. The psychiatric assessment included the Outcome Inventory 21 (OI-21), Inner Strength-Based Inventory (i-SBI), Revised Thai version of the Multidimensional Scale of Perceived Social Support (r-MSPSS), Zuckerman, Kuhlman and Aluya Personality Questionnaire (ZKA-40) and the Thai version of the 10-item Perceived Stress Scale (T-PSS-10). All measurements were conducted using the Thai versions. The Scale for the Assessment and Rating of Ataxia (SARA), used to assess ataxic severity, was administered by neurologist investigators, while research assistants administered the Montreal Cognitive Assessment (MoCA) and the Thai version of the Pittsburgh Sleep Quality Index (PSQI).

### 2.3. Measurements

#### 2.3.1. Outcome Inventory 21 (OI-21)

The OI-21 is a self-report scale assessing four main symptoms commonly found in patients with ALS: depression, anxiety, somatization and interpersonal difficulty. In this study, this questionnaire was used to assess depression and anxiety. The questionnaire consists of 21 questions on a Likert scale. Patients rated the items that most closely matched their feelings on a scale from 0 (not at all) to 4 (almost always), with a Cronbach’s alpha of 0.92. The Cronbach’s alpha for each subscale was 0.82 for anxiety and 0.87 for depression. The OI-depression subscale has a cut-off value of 7, meaning that a score greater than or equal to 7 indicates depression, with sensitivity and specificity values of 82.72 and 78.35, respectively [20].

#### 2.3.2. Inner-Strength-Based Inventory (i-SBI)

The i-SBI is a self-report scale measuring ten inner strengths considered characteristics of positive psychology including generosity, morality, mindfulness/meditation, wisdom, perseverance, patience, endurance, truthfulness, determination, loving-kindness and equanimity. This questionnaire uses a Likert scale consisting of 10 questions, where patients rate the items that best match their feelings from 1 to 5. The reliability coefficient is 0.86, and the item reliability is 0.99, and the results are divided into scores as follows: low level of strength (10–20), average strength (21–39) and high level of strength (40–50) [21].

#### 2.3.3. Revised Thai Version of the Multidimensional Scale of Perceived Social Support (r-MSPSS)

The r-MSPSS measures perceived social support across 3 factors: family, friends and significant others. This Likert scale involved 12 questions, where patients rated the items most closely matching their feelings. A score of 0 represents strong disagreement, while a score of 7 is the maximum. The Cronbach’s alpha for this scale is 0.92. The total score for the 12 questions ranges from 0 to 84, divided into three levels: low (12–36), medium (37–60) and high (61–84) [22].

#### 2.3.4. Zuckerman, Kuhlman, and Aluya Personality Questionnaire (ZKA-40)

The ZKA-40 is a self-report scale measuring five personality traits according to the alternative five-factor model (AFFM), namely aggression (AG), sensation seeking (SS), activity (AC), extraversion (EX) and neuroticism (NE). The scale consists of 40 questions, with 8 items for each personality trait. The Cronbach’s alpha for each domain was 0.829 for aggression, 0.817 for sensation seeking, 0.75 for activity, 0.831 for extraversion and 0.854 for neuroticism. The interpretation of the scores is as follows: a standard score in the range of 40–60 is considered normal, while a score of 60 and above is considered high. A score below 40 is considered low. The higher or lower the standard score, the more pronounced the characteristics represented by that scale [23].

#### 2.3.5. Thai Version of the 10-Item Perceived Stress Scale (T-PSS)

The T-PSS is a self-report scale for measuring stress. This questionnaire uses a Likert scale and has a Cronbach’s alpha of 0.85. It consists of 10 questions, each scored from 0 to 4, where 0 means “not at all” and 4 means “very often”. The scores are categorized as follows: low stress (0–13), moderate stress (14–26) and high stress (27–40) [24].

#### 2.3.6. The Scale for the Assessment and Rating of Ataxia (SARA)

The SARA assesses ataxic severity, consisting of 8 dimensions: gait, stance, sitting, speech, finger-chase test, nose-finger test, fast alternating movements and the heel–shin test. The Cronbach’s alpha is 0.92, and scoring ranges from 0 to 40 points, where 0 indicates no ataxia and 40 represents the most severe ataxia [25]. A comparison of SARA scores from face-to-face examinations with those obtained via remote video showed no significant differences between the two methods [26].

#### 2.3.7. Thai Version of Pittsburgh Sleep Quality Index (PSIQ)

The PSIQ is a self-report scale for assessing sleep quality. This questionnaire uses a Likert scale and has a Cronbach’s alpha of 0.837. The tool is divided in 7 components: sleep quality, sleep onset latency, sleep duration, sleep efficiency, sleep disturbances, hypnotic drugs and daytime dysfunction. The results are interpreted by combining the scores from all seven components, with total scores ranging from 0 to 21 points. A total score of 5 or lower indicates good sleep quality, while a score above 5 indicates poor sleep quality [27].

#### 2.3.8. Montreal Cognitive Assessment (MoCA) Thai Version

MoCA is a screening tool for cognitive impairment, with a Cronbach’s alpha of 0.914. The tool assesses various functions including attention, executive function, naming, memory, language, visuospatial function, delayed recall, abstraction and orientation. The maximum score is 30 points, and a score of 25 or higher is considered normal [28]. A comparison of the MoCA test conducted in person and via videoconference showed no significant differences in the results [29,30].

### 2.4. Statistical Analysis

The data analysis followed a systematic approach, as outlined below.

1. Descriptive Statistics: We began by calculating frequencies and percentages for categorical variables (e.g., sociodemographic data) and means and standard deviations for continuous variables, e.g., OI-21, i-SBI, ZKA-40, T-PSS-10, SARA and MoCA. This provided a comprehensive overview of the sample and the prevalence of depression.
2. Pearson’s Correlation: Pearson’s correlation was used for categorial or ordinal variables, e.g., sex, marital status and education. A bivariate association between OI-depression, OI-anxiety and positive mental health scores (r-MSPSS and i-SBI) and negative mental health scores (OI-21, T-PSS and ZKA-40) were examined using Pearson’s correlation.
3. Bootstrapping: To enhance the accuracy of our results, we applied a bootstrapping method (1000 resamples) to calculate more reliable confidence intervals for the correlations, especially given the sample size and nonnormal distribution.
4. Significance Testing: All analyses were conducted with a significance threshold of *p* < 0.05 to determine the statistical significance of the associations.
5. Software: All statistical analyses were performed using IBM SPSS, Version 29, (IBM Corp., Armonk, NY, USA).

## 3. Results

We used descriptive statistics to summarize the distribution of key variables. Of the eleven patients included in this study, six were women. The participant’s average age was 50.27 ± 14.485 years. The mean age at onset and age at diagnosis was 32.82 ± 15.626 and 35.91 ± 16.115 years old, respectively. The most common comorbidity was hypertension. More than half of patients were being treated for depression, and the prevalence of depression was 27.27%. The means of the OI-21, I-SBI, r-MPSS, Personality trait (T-score), T-PSS, SARA, PSIQ and MoCA scores are shown in Table 1.

Table 2 presents the results of factors associated with depression scores. Pearson’s correlation was conducted to explore the strength and direction of sociodemographic and clinical variables and depression. Insufficient income showed positive correlation with depression scores (*r* = 0.687, *p*-value 0.019 and 95% CI 0.115 to 0.905). Moreover, age at onset, age at diagnosis and age at treatment also showed a negative correlation with depression (*r* = −0.602, *p*-value 0.050, 95% CI −0.879 to 0.027 and *r* = −0.683, *p*-value 0.020, 95% CI −0.904 to −0.108, respectively).

The results of the correlation between mental health factors and OI-depression scores are shown in Table 3. Pearson’s correlation was conducted to explore the strength and direction of mental health factors and depression. Factors positively associated with the OI-depression score were OI-anxiety, perceived stress and personality traits including aggression, activity and neuroticism. However, perceived support from family and total inner strengths negatively correlated with OI-depression.

## 4. Discussion

This study investigated the relationship between mental health issues including depression and associated factors among patients with SCA. Although over one half of the participants reported a history of psychiatric treatment, the prevalence of depression was 27.27%, consistent with other related studies [9,10].

Compared with the mean score of OI-depression found in the general population, the scores among this sample were significantly higher [20]. This implies that the patients with SCA in this study experienced more depressive symptoms than the general population. This finding is not unexpected, as SCA is a progressive, debilitating condition that can significantly impact physical function, independence and overall quality of life [9].

Several factors were associated with depression including insufficient income, younger age at disease onset/diagnosis/treatment, lower levels of perceived social support and personality traits including aggression, activity and neuroticism. Additionally, higher levels of anxiety and perceived stress were also associated with increased depression. Numerous studies support these factors, and each will be explained below.

Patients with an earlier age of onset face longer periods of disability and dependence, leading to a higher prevalence of depression. Additionally, insufficient income exacerbates the challenges of living with SCA, contributing to increased depression. Importantly, this study confirmed the hypothesis that inner strengths and perceived social support, particularly from family members, are negatively correlated with depression and anxiety scores. Both inner strengths and perceived family support could be seen as protective factors for depression among SCA. This could be the first study to explore the positive factors among patients with SCA. The negative correlation with the inner strengths suggested it could be considered a protective factor.

Perceived Social Support and Depression among patients with SCA

As shown in Table 3, perceived family support significantly and negatively correlated to depression scores. This aligns with related research on mental health factors associated with depression. For instance, studies have shown that perceived social support from family members and friends is associated with reduced depression among patients with a major depressive disorder [11]. Similarly, research concerning patients with ALS found a negative correlation between depression and social support and environmental resources [12]. For patients with SCA, this association between family support and reduced depression in SCA may be due to several factors. First, family support is particularly crucial due to the inevitable progression towards dependence. Physical and emotional support from family members, leading to a sense of being supported, empowers patients to better cope with life’s challenges, potentially reducing the risk of depression. Second, being part of a family fosters a sense of belonging and reduces feelings of loneliness, a major risk factor for depression [31].

Inner Strengths and Depression among patients with SCA

Our findings suggest that inner strengths play a significant role in mitigating depression among individuals with SCA. As shown in Table 3, total inner strength scores negatively correlated to depression scores. This indicates that individuals with higher levels of inner strength tend to experience fewer depressive symptoms. In the context of SCA, this progressive neurological condition presents significant physical and emotional challenges. Inner strengths are crucial for maintaining mental well-being, not only by buffering the impact of stress but also by fostering positive emotions and behavioral characteristics that enable patients to better cope with the stressors related to their condition. Moreover, certain inner strengths, such as loving-kindness and compassion, promote stronger interpersonal relationships and reduce feelings of loneliness, aligning with cited findings. This study’s results are consistent with research emphasizing the important role of inner strengths in chronic illness and promoting overall well-being [32].

Personality Traits, Anxiety and Depression among patients with SCA

Our findings indicate a positive association between certain personality traits and anxiety and the depression scores among patients with SCA (Table 3). Specifically, neuroticism and aggression showed positive correlations with depression symptoms. This suggests that patients with SCA exhibiting higher levels of neuroticism and aggression may be particularly vulnerable to experiencing depression. Both biological and psychological factors can explain this. Emotional dysregulation among patients with SCA is partly due to neurological degeneration. The cerebellum has extensive connections with the frontal lobes and brainstem, and cerebellar degeneration can lead to cognitive deficits and emotional disturbances, known as cerebellar cognitive affective syndrome. This syndrome can manifest as depression, anxiety and aggression. Brain atrophy can cause shrinkage of certain brain regions including the frontal lobe, which is involved in planning, decision-making and impulse control [9].

In addition to biological factors, the condition could be due to emotional instability and difficulty coping with stress that characterizes neuroticism. The symptoms of SCA can be a significant source of stress due to the loss of motor control and the challenges of living with a chronic illness. For patients with neurotic tendencies, these stressors may be even more difficult to manage, further increasing their risk of depression. This is consistent with related research findings in the general population [33].

Additionally, neuroticism is associated with depression in SCA patients, which can be explained by the frustration and anger that may arise from living with a progressively debilitating disease. If these emotions are not managed effectively, they can manifest as aggression or, alternatively, contribute to depression when turned inward [33]. The loss of physical control and independence associated with SCA can be very distressing and may lead to feelings of helplessness and hopelessness, which are key features of depression. In some cases, aggression may be an attempt to regain a sense of control [34].

An aggressive personality trait is also positively associated with anxiety among patients with SCA. In addition to the biological factors explained earlier, patients with this personality trait tend to express aggression when provoked or slighted. Because their disability may prevent them from expressing their emotions as they would like, this can lead to frustration and anxiety. Furthermore, motor symptoms in SCA can lead to decreased activity levels, which can be particularly challenging for individuals with an active personality trait who naturally enjoy staying busy and engaged. This can lead to frustration, boredom and a sense of loss, potentially contributing to depression. Furthermore, motor symptoms in SCA can lead to decreased activity levels, which can be particularly challenging for individuals with an active personality trait who naturally enjoy staying busy and engaged. This can lead to frustration, boredom and a sense of loss, potentially contributing to depression.

Perceived Stress and Depression among patients with SCA

Our study found a positive correlation between perceived stress and depression among patients with SCA. This suggests that individuals with SCA experiencing higher levels of stress tend to also report more severe depressive symptoms. This finding is consistent with research in the general population, showing a clear link between perceived stress and depression [34]. Stress causes depression, and depression can also increase perceived stress. This creates a vicious cycle where each can worsen the other [34]. In SCA, stressors are often linked to both the physical symptoms of the disease and the psychological burdens of coping with a chronic, debilitating condition. The unpredictability of disease progression and the daily challenges faced by patients with SCA, e.g., difficulty with mobility and daily tasks, can increase perceived stress, making it harder to manage emotional well-being. The experience of stress is further compounded by the frustration of dealing with a progressively worsening condition, which, for many patients, can evoke feelings of helplessness and anxiety.

### 4.1. Implication of This Study and Future Research

Our findings have important implications for clinical practice. They highlight the need for healthcare professionals to assess and address mental health concerns, particularly depression and anxiety, among patients with SCA. The early identification of and intervention for depression and anxiety are crucial. Healthcare professionals should routinely screen for depression and anxiety among patients with SCA using validated tools and clinical interviews, paying special attention to those facing financial difficulties, as income insufficiency is strongly associated with depression.

Specific factors to address:

- Social Support: Encourage family involvement in care, as family support is key in reducing depression. Family therapy or support groups may be helpful.
- Stress Management: Incorporate stress management techniques, such as meditation [35], to address the correlation between stress and depression.
- Personality Traits: Targeted interventions for patients with high neuroticism or aggression may improve emotional regulation and coping skills.

Healthcare professionals can also educate patients on the mental health impacts of SCA and encourage help-seeking behaviors by providing resources for support and treatment.

Future research should explore the effectiveness of targeted interventions aimed at enhancing inner strengths, reducing perceived stress and strengthening social support networks in improving the mental health of patients with SCA. Investigating the specific mechanisms through which these factors influence mental health in SCA would also be valuable.

### 4.2. Strengths and Limitations

This research encountered the limitations explained below.

1. Genetic Confirmation: Not all patients were confirmed to have SCA through genetic testing. Most underwent testing only for common types of SCA. If the results were negative, further testing for other SCA types was often not pursued due to cost constraints. However, clinical diagnosis by a neurologist and MRI findings consistent with SCA were used in these cases. This lack of genetic confirmation represents an important limitation of the study, as it is possible that participants may have had other, less common forms of ataxia, affecting the interpretation and generalizability of the findings. Future research should prioritize genetic confirmation to accurately diagnose SCA subtypes and enhance the validity of results.
2. Prior Psychiatric Treatment: Patients participating in this study were already receiving psychiatric medication, which may have affected their OI-depression scores, potentially leading to lower scores than they would have otherwise had.
3. Small Sample Size: The small sample size limits the generalizability of these findings. While our results offer valuable insights, they may not represent all patients with SCA. Future research with larger samples is needed to confirm these findings.
4. Cross-Sectional Design: The study’s cross-sectional design prevents us from drawing causal conclusions about the relationships between the variables.
5. A heavy reliance on self-report measurements: This study relied on self-reported data, which can be subject to response bias. Participants may have recalled difficulties or answered questions in a way they felt was more desirable, potentially affecting the accuracy of our findings. While we used validated questionnaires with established psychometric properties and ensured confidentiality to minimize bias, it cannot be entirely ruled out.

Despite these limitations, this research focuses on a specific population, examining mental health factors in SCA patients, a group relatively understudied in terms of mental health. The study investigates various mental health factors, both positive, e.g., social support and inner strengths, and negative, e.g., perceived stress and personality traits. This provides a more comprehensive understanding of the factors contributing to depression and anxiety in patients with SCA.

Finally, the results highlight several factors significantly associated with depression and anxiety in SCA such as income, age at onset/diagnosis/treatment, perceived social support and personality traits. These findings can inform clinical practice and future research.

## 5. Conclusions

This study provides insights into the relationship between positive and negative mental health factors associated with depression among patients with SCA. Our findings highlight the importance of conducting comprehensive mental health assessments and implementing targeted interventions for this population. Emphasis should be placed on enhancing inner strengths and fostering supportive relationships. Furthermore, efforts to reduce perceived stress and address specific personality traits are essential. However, given the small sample size, these results should be interpreted cautiously. Future research with larger, randomly selected samples is needed to confirm the robustness and generalizability of these findings.

## Figures and Tables

**Table 1:** Sociodemographic characteristics and clinical data.

Variables	All (*n* = 11)
Age (years)	50.27 ± 14.485
Sex, female	6 (54.5%)
Marital Status	
Single	4 (36.4%)
Married and lived with their partner	5(45.5%)
Married but not lived with their partner	1 (9.1%)
Widowed	1 (9.1%)
Having children, having	7 (63.6%)
Income, sufficient	7 (63.6%)
Educational level	
Primary education	3 (27.3%)
Vocational education	3 (27.3%)
Higher education	5 (45.5%)
Co-morbidities	
None	4 (25%)
Hypertension	4 (25%)
DM	2 (12.5%)
Other	6 (37.5%)
Received psychiatric treatment, depression	6 (54.5%)
Psychiatric disease in the family	
None	9 (81.8%)
Depression	2 (18.2%)
Having a depression	3 (27.27%)
Age at onset	32.82 ± 15.626
Age at diagnosis	35.91 ± 16.115
Age of treatment	35.91 ± 16.115
OI-21	
Depression	4.64 ± 3.88
Anxiety	6.55 ± 5.989
Interpersonal difficulty	4.36 ± 4.007
Somatization	5.64 ± 3.88
Total	21.18 ± 16.161
i-SBI	32.09 ± 3.961
r-MSPSS	62.55 ± 10.976
Personality trait (T-score)	
Aggression (AG)	49.55 ± 19.557
Sensation seeking (SS)	46.36 ± 8.835
Activity (AC)	48.09 ± 10.663
Extraversion (EX)	50.73 ± 8.247
Neuroticism (NE)	46.45 ± 13.845
T-PSS	17.55 ± 6.609
Scale for the assessment and rating of ataxia	19.82 ± 10.925
Pittsburgh sleep quality index	23.91 ± 11.995
Montreal Cognitive Assessment	18.45 ± 7.866

**Table 2:** Correlation between sociodemographic and clinical variables and OI-depression score.

Variables	Correlation Coefficients (*r*)	Bootstrapping Bias-Corrected 95% CI		*p*-Value
		Lower	Upper	
Sociodemographic variables				
Age	−0.322	−0.766	0.359	0.334
Sex, female	0.009	−0.594	0.605	0.979
Income, insufficient	0.687	0.115	0.905	0.019
Received psychiatric treatment, depression	0.256	−0.417	0.736	0.448
Clinical variables				
Age at onset	−0.602	−0.876	0.027	0.050
Age at diagnosis	−0.683	−0.904	−0.108	0.020
Age of treatment	−0.683	−0.904	−0.108	0.020
Scale for the assessment and rating of ataxia	0.347	−0.334	0.777	0.295
Pittsburgh sleep quality index	0.364	−0.318	0.784	0.270
Montreal cognitive assessment	−0.037	−0.622	0.577	0.915

**Table 3:** Correlation between mental health factors and depression symptoms.

Variables	Correlation Coefficients (*r*)	Bootstrapping Bias-Corrected 95% CI		*p*-Value
		Lower	Upper	
Outcome inventory-21				
OI-21 Anxiety	0.887	0.586	0.968	<0.001
OI-21 Interpersonal difficulty	0.723	0.183	0.917	0.012
OI-21 Somatization	0.548	−0.104	0.857	0.081
OI-21 Total	0.880	0.564	0.966	<0.001
Perceived stress				
T-PSS	0.781	0.305	0.936	0.005
Personality				
Aggression	0.730	0.197	0.920	0.011
Sensation seeking	0.060	−0.562	0.635	0.862
Activity	0.651	0.052	0.893	0.030
Extraversion	−0.466	−0.826	0.208	0.149
Neuroticism	0.800	0.351	0.942	0.003
Perceived social support				
r-MSPSS Significant other	0.054	−0.566	0.632	0.874
r-MSPSS Friends	0.036	−0.578	0.621	0.917
r-MSPSS Family	−0.880	−0.966	−0.564	<0.001
r-MSPSS Total	−0.441	−0.816	0.237	0.175
Inner strength				
i-SBI	−0.700	−0.910	−0.139	0.016

## Data Availability

The datasets generated and/or analyzed during the current study are not publicly available due to ethics approval but are available from the corresponding author on reasonable request.
